# Manipulation of Skyrmion Motion Dynamics for Logical Device Application Mediated by Inhomogeneous Magnetic Anisotropy

**DOI:** 10.3390/nano12020278

**Published:** 2022-01-16

**Authors:** Jia-Qiang Lin, Ji-Pei Chen, Zhen-Yu Tan, Yuan Chen, Zhi-Feng Chen, Wen-An Li, Xing-Sen Gao, Jun-Ming Liu

**Affiliations:** 1 School of Physics and Materials Science and Research Center for Advanced Information Materials, Guangzhou University, Guangzhou 510006, China; 2111919015@e.gzhu.edu.cn (J.-Q.L.); 2112119018@e.gzhu.edu.cn (Z.-Y.T.); chenyuan@gzhu.edu.cn (Y.C.); chenzf@gzhu.edu.cn (Z.-F.C.); liwa@gzhu.edu.cn (W.-A.L.); 2 Guangdong Provincial Key Laboratory of Quantum Engineering and Quantum Materials and Institute for Advanced Materials, South China Normal University, Guangzhou 510006, China; liujm@nju.edu.cn; 3 Laboratory of Solid State Microstructures and Innovative Center of Advanced Microstructures, Nanjing University, Nanjing 210093, China

**Keywords:** magnetic skyrmion, inhomogeneous perpendicular magnetic anisotropy, logic devices, micromagnetic simulation

## Abstract

Magnetic skyrmions are promising potential information carriers for future spintronic devices owing to their nanoscale size, non-volatility and high mobility. In this work, we demonstrate the controlled manipulation of skyrmion motion and its implementation in a new concept of racetrack logical device by introducing an inhomogeneous perpendicular magnetic anisotropy (PMA) via micromagnetic simulation. Here, the inhomogeneous PMA can be introduced by a capping nano-island that serves as a tunable potential barriers/well which can effectively modulate the size and shape of isolated skyrmion. Using the inhomogeneous PMA in skyrmion-based racetrack enables the manipulation of skyrmion motion behaviors, for instance, blocking, trapping or allowing passing the injected skyrmion. In addition, the skyrmion trapping operation can be further exploited in developing special designed racetrack devices with logic AND and NOT, wherein a set of logic AND operations can be realized via skyrmion–skyrmion repulsion between two skyrmions. These results indicate an effective method for tailoring the skyrmion structures and motion behaviors by using inhomogeneous PMA, which further provide a new pathway to all-electric skyrmion-based memory and logic devices.

## 1. Introduction

A magnetic skyrmion is a topologically stable configuration often observed in chiral magnets with broken inversion symmetry. The nanoscale skyrmion exhibits topologically stable particle-like behavior, and it can be efficiently created, annihilated and moved by ultra-low critical current densities. These characters make it promising candidate as information carrier for next-generation of spintronic devices [1,2,3,4,5,6]. It has thus aroused intense research efforts in recent years, leading to a series of breakthrough achievements in manipulation of skyrmion states, via, e.g., injected spin-polarized currents, or external electric-field [7,8,9,10,11,12,13,14,15,16]. These achievements underpin a wide range of emerging skyrmion-based spintronic devices, including racetrack memories, high-density magnetic random access memories, logic gates, etc. [1,2,3,4,5,6,7,8,9,10], which hold advantages for all-electrical control and energy-efficient fashion [2,3].

It is well known that the skyrmion structure usually arises as a result of energy competition among the exchange energy, Dzyaloshinskii–Moriya interaction (DMI), demagnetization energy, magnetic anisotropy and Zeeman energy [8]. Previous studies demonstrated that various types of anisotropy terms (e.g., perpendicular magnetic anisotropy (PMA), easy-plane magnetocrystalline anisotropy and cubic anisotropy) may play a prominent role in modulating the skyrmion structures and enhancing their stability [17,18,19,20,21,22,23,24,25]. For instance, PMA can help create and stabilize skyrmions, regulate their structures [13,17,18,26,27], and even enables the stabilization of skyrmion at zero magnetic field in confined nanostructures [8].

So far, most of the investigations regarding the effects of PMA on skyrmions focused on homogenous PMA systems. Recently, inhomogeneous PMA introduced by using capping layer [28,29,30] or a voltage-controlled magnetic anisotropy (VCMA) gate [31] has also attracted more and more interests. Such a nonuniform PMA can serve as potential well or barriers to control the local dynamic behavior of skyrmion, e.g., pinning or depinning of skyrmion, which shows promising features for applications in skyrmion transistors, memories, logic gates, etc. [30,32,33,34,35,36,37]. To explore the intriguing magnetic devices for multifunctional logic applications, most investigations were carried out in the multi-channel and multi-network circuits [34,35,36,37]. For example, a recent study proposed a technique based on reversible computing and conservative logic for achieving large-scale circuits composed of cascaded logic gates, by utilizing the VCMA scheme [34]. However, to date, how to design the skyrmion-based logic device in a single-channel circuit (e.g., a straight nanotrack) with inhomogeneous PMA is still an open question. Therefore, it is crucial to explore the potential applications for expanding the functions of skyrmionic devices in this field. To achieve this goal, it is also of great essential for manipulation of skyrmion structures and their dynamic behaviors.

Inspired by this motivation, we studied the manipulation of skyrmion structure and dynamic behaviors mediated by local inhomogeneous PMA in heterostructures with center capping nano-island, by using micromagnetic simulation. We first examine the effect of inhomogeneous PMA distribution on the structure and dynamic behaviors of isolated skyrmion confined in nanostructures and nanotracks. Based on these findings, we proposed the skyrmion-based logical devices with inhomogeneous PMA which can successfully achieve the logic AND and NOT operations by taking into account of the complicated dynamical behaviors of two interacting skyrmion movements in nanotracks. The findings demonstrate an effective approach for tailoring skyrmion structures and controlling their dynamics behaviors via inhomogeneous PMA, which also provide new routes to develop more skyrmion-based spintronic devices.

## 2. Model and Simulation Methods

**Figure 1:**
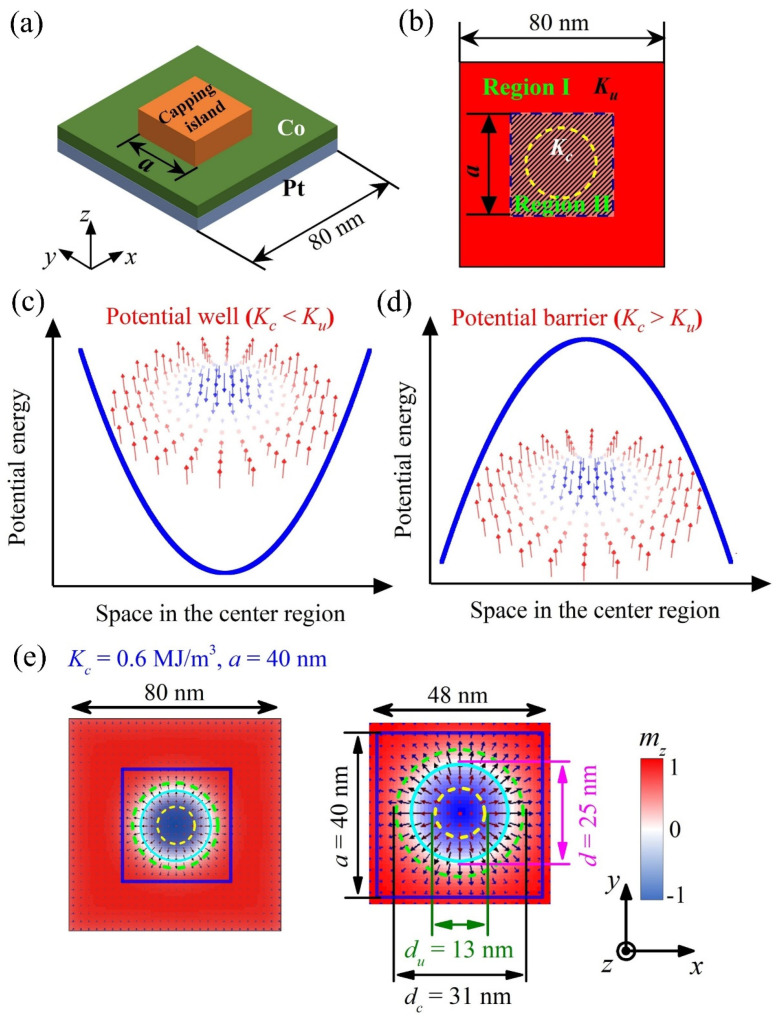
(**a**) Sketch of magnetic heterostructure of a square-shaped capping nano-island built on a square-shaped ultrathin Co/Pt film. (**b**) Schematic view of inhomogeneous PMA distribution on the *xy*-plane, in which the PMA strength (*K_c_*) in capping island (Region II) is variable by the capping materials, and the PMA strength (*K_u_*) in the outer ring of the Co/Pt film (Region I) is fixed with *K_u_* = 0.8 MJ/m^3^. The inserted yellow dashed circle with diameter of 30 nm represents spin-polarized current injection (*p* = 0.4) in the central region. (**c**,**d**) The nucleation of skyrmion in panel (**c**) potential well for *K_c_ < K_u_* or (**d**) potential barrier for *K_c_ > K_u_*. (**e**) The isolated skyrmion with diameter *d* under the inhomogeneous PMA distribution of *K_c_* = 0.6 MJ/m^3^ and *a* = 40 nm, with the enlarge image of the skyrmion structure shown at the right panel. Insert shows the color scale for the *z*-component of magnetization configurations *m_z_*. The capping region is enclosed by blue lines. The diameters *d*, *d*_c_ and *d_u_* are marked by the cyan solid, green dashed, and yellow dashed circles, respectively.

In this work, we studied the manipulation of skyrmion mediated by local inhomogeneous PMA in the heterostructures. We proposed an architecture of a capping nano-island fabricated on the top surface of an ultrathin Co/Pt film, in which the PMA strength (*K_c_*) of capping island (Region II) can be largely modified by different capping materials [28,29,30], as schematically shown in Figure 1a. Here, the desired *K_c_* can be manufactured by choosing a proper capping material with modified material parameters, e.g., strain and sample thickness. The PMA strength (*K_u_*) in the outer ring of the Co/Pt film (Region I, out of capping region) was considered to be a fixed strength of *K_u_* = 0.8 MJ/m^3^ [8]. Therefore, the PMAs in Region I and Region II are different, which induces an inhomogeneous PMA distribution in the Co/Pt film, as presented in Figure 1b.

The magnetic state in the Co/Pt film is usually dependent on the total free energy (*E*), including Heisenberg exchange energy, DMI contribution, PMA energy and demagnetization energy, which is written as [8]

(1) $$\begin{array}{l} E=\int dr[A_{ex}{\left(\nabla \cdot m\right)}^{2}+D\left(m_{z}\frac{\partial m_{x}}{\partial x}-m_{x}\frac{\partial m_{z}}{\partial x}+m_{z}\frac{\partial m_{y}}{\partial y}-m_{y}\frac{\partial m_{z}}{\partial y}\right)\\ \;-K\left(m\cdot e_{z}\right)-\frac{\mu_{0}}{2}M_{s}m\cdot H_{d}] \end{array},$$

where *A_ex_* and *K* are the ferromagnetic exchange and effective anisotropy energy constants, respectively. **H***_d_* is the magnetostatic self-interaction fields, and *D* is the DMI constant. ***m*** = **M**/M_s_ = (*m_x_*, *m_y_*, *m_z_*) is the normalized magnetization vector with M_s_ the saturation magnetization. The PMA easy-axis is along the ±*z*-axis.

To investigate the dynamics of the magnetic structures driven by the spin-polarized current, the simulation was conducted by employing a three-dimensional Object-Oriented Micromagnetic Framework (OOMMF) [38], in which time-dependent magnetization dynamics was computed by solving the Landau–Lifshitz–Gilbert (LLG) equation [39,40]:

(2) $$\frac{dm}{dt}=-\gamma m\times H_{eff}+\alpha m\times \frac{dm}{dt}+T,$$

where the first and second terms on the right side of the equation describe the gyromagnetic precession and the Gilbert damping respectively, and the third term **T** denotes the spin transfer torque (STT) due to the spin-polarized current. ***H****_eff_* = −(1/*μ*_0_M_s_)∂*E*/∂***m*** is the effective field, *γ* is the Gilbert gyromagnetic ratio, and *α* is the damping coefficient.

For creating the isolated skyrmions in the magnetic nanostructures, we inject a spin polarization current perpendicular the plane (CPP), with the current-induced spin transfer torque $T_{\mathrm{CPP}}$ written as [8]

(3) $$T_{\mathrm{CPP}}=\frac{u}{t}m\times m_{p}\times m,$$

where *u* = *γ*(*ℏj*P*/*2*e*M_s_) is the Slonczewski torque coefficient, *t* is the film thickness of ferromagnetic layer, *j* is the current density, *e* is the elementary charge, p is the polarization rate and ***m***_p_ is the electron polarization direction.

For simulation of the skyrmion dynamics induced by the current-in-plane (CIP) injection along *x*-aixs in the nanotrack, the corresponding spin transfer torque **T**_CIP_ is given by the following form [8,10]:

(4) $$T_{\mathrm{CIP}}=um\times (m\times \frac{\partial m}{\partial x})+\beta um\times \frac{\partial m}{\partial x},$$

where the first and second terms represent the coupling between magnetic moments and spin-polarized current *j* via the spin transfer torque and via the non-adiabatic effects respectively, with β the non-adiabaticity factor.

In the simulations, we considered that the Co/Pt bilayers contain a 0.4-nm-thick cobalt film. The typical parameters for studying the current-induced skyrmion dynamics were adopted as [8]: the spontaneous magnetization M_s_ = 580 KA/m, exchange constant *A_ex_* = 15 pJ/m, DMI constant *D* = 3 mJ/m^2^, gyromagnetic ratio *γ* = 2.211 × 10^5^ m/(A·s), damping coefficient *α* = 0.3, non-adiabaticity factor *β* = 0.3 and polarization rate p = 0.4. The nanomagnets are divided into unit cells with cell size of 1 × 1 × 0.4 nm^3^ for the simulations.

## 3. Results and Discussion

### 3.1. Nucleation of Skyrmion in Square-Shaped Nanostructures with Inhomogeneous PMA

We first investigated the nucleation of skyrmion in square-shaped Co/Pt nanostructures mediated by the inhomogeneous PMA distribution, as presented in Figure 1b. The simulations were carried out on a heterostructure of a square-shaped capping nano-island (length *a*) fabricated on the top surface of a square-shaped ultrathin Co/Pt film (length 80 nm). Here, *K_c_* < *K_u_* and *K_c_* > *K_u_* represent central potential wells and central potential barriers in the nanostructures, respectively (see Figure 1c,d). We may consider that the absolute difference of anisotropy |*K_c_* − *K_u_*| quantifies the depth/height of potential wells/barriers, and the size *a* of capping nano-island characterizes the area of central potential wells/barriers. In this sense, one may understand that the concept of inhomogeneous PMA distribution, which performs as potential barriers/wells in the nanostructure, with their height/depth and area tunable by the PMA and size of the capping region in the heterostructure. In contrast to the VCMA gate, the size of capping nano-island here cannot be changed on demand once the sample is produced, which reduces the tunability of the well/barrier. However, circuits made of heterostructures with capping nano-island can serve as specific functional devices without requiring external control which reduce circuit complexity.

Here, we focus on the effect of inhomogeneous PMA on the skyrmion size, which is characterized by the diameter of skyrmion *d* defined as the diameter of the circle with *m_z_* = 0 in the skyrmion configurations [41]. For the skyrmion nucleation in the nanostructures, the simulation starts from an initial ferromagnetic state (*m_z_* = +1, colored red), and then a 1 ns long CPP-type current pulse with *j* = 1.0 × 10^9^ A/cm^2^ is applied locally perpendicular to the Co/Pt film on the central 30 nm region. Next, the system is relaxed under *j* = 0.0, and subsequently the isolated skyrmion structure is obtained as the equilibrium state.

Figure 1e presents a nucleated isolated skyrmion with diameter *d* in the nanostructure under inhomogeneous PMA distribution as *K_c_* = 0.6 MJ/m^3^ and *a* = 40 nm. In order to elucidate the effect of inhomogeneous PMA on the skyrmion size, we calculated the special diameter of isolated skyrmion *d_c_* or *d_u_*, in which a homogeneous PMA with anisotropy of *K* = *K_c_* or *K_u_* is set over the whole square nanostructure. Generally, the skyrmion diameter reduces with increasing the homogeneous PMA strength [17,18,26], and the simulated results showed that *d_u_* = 13 nm for *K* = *K_u_* = 0.8 MJ/m^3^ and *d_c_* = 31 nm for *K* = *K_c_* = 0.6 MJ/m^3^ in the homogeneous PMA, as marked in Figure 1e. Remarkably, the inhomogeneous PMA distribution here changes the energy distribution over the nanostructure, making the skyrmion diameter *d* greatly different from *d_u_* or *d_c_* in the homogeneous PMA.

**Figure 2:**
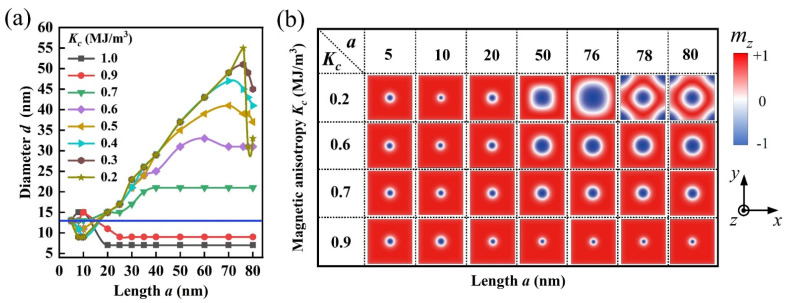
Effect of inhomogeneous PMA distribution on the structure of skyrmion. (**a**) Plots of skyrmion diameter *d* as a function of *a* under various fixed *K_c_*. Here the blue straight line marks the case of homogeneous PMA with *K_c_* = 0.8 MJ/m^3^. (**b**) The spatial profile of the *z*-component of some typical magnetization configurations in the nanostructures under various *a* and *K_c_*. Here all the shown areas are 80 nm × 80 nm in size for the nanostructures.

To proceed, we calculated the dependences of skyrmion diameter *d* on the variables *a* and *K_c_*, with the simulated results given in Figure 2. Next, we discuss the simulated results according to the nano-island size *a*, which is divided into three regions as: small *a* (~5 nm ≤ *a* ≤ ~20 nm), medium *a* (~20 nm ≤ *a* ≤ ~70 nm), and large *a* (~70 nm ≤ *a* ≤ ~80 nm).

It was found in Figure 2a that *d* is small and falls within ~6 nm ≤ *d* ≤ ~15 nm in the small *a* case. For medium *a*, *d* in the case of central potential wells (*K_c_* < *K_u_*) is always larger than that in the case of central potential barriers (*K_c_* > *K_u_*). This is because that the anisotropy energy in capping island decreases with *K_c_*, and it becomes weaker than the demagnetization energy in the case of small *K_c_* = 0.2 MJ/m^3^. This results in a crossover from perpendicular magnetization (i.e., easy-axis magnetization) to in-plane magnetization (i.e., easy-plane magnetization) with decreasing *K_c_*, and thus the expansion of skyrmion structures. Note that the skyrmion diameter *d* increases with *a* for the cases of central potential wells, while *d* keeps nearly constant for the cases of central potential barriers. This implies that the central potential wells are suited for generating the large-sized skyrmion (see the maximal diameter *d*_max_ ~55 nm at *K_c_* = 0.2 MJ/m^3^), while the central potential barriers are applicable for producing the small-sized skyrmion (see the minimal diameter *d*_min_ ~7 nm at *K_c_* = 1.0 MJ/m^3^).

In addition, for the large *a* case, it was noted in Figure 2a that *d* decreases with increasing *a* at 0.2 MJ/m^3^ ≤ *K_c_* ≤ 0.6 MJ/m^3^. This tendency especially occurs for the skyrmion with large size, which manifests the restriction of nanostructure boundary on the enlargement of skyrmion size. For example, one may see the special case of *K_c_* = 0.2 MJ/m^3^ in Figure 2b that, the diameter *d* is ~55 nm at *a* = 76 nm, while it shrinks sharply to be ~32 nm at *a* = 78 nm with the skyrmion becoming surrounded by four Néel-type magnetic kinks (domain walls) [42]. This is because that the skyrmion structures cannot expand beyond the nanostructure, and it thus shrinks as a result of the boundary constrictions of the nanostructure.

From the simulation results and analyses above, one may tailor the skyrmion structure on demand by introducing different energy potential wells or energy potential barriers into the nanostructure via the inhomogeneous PMA distribution. To preserve the rotational symmetry of skyrmion, many studies focused on isolated skyrmions confined in circular nanodisks [8,13], and it was demonstrated that the isolated skyrmions can be stabilized by using inhomogeneous PMA in disk-shaped heterostructure with circle-shaped capping layer [30]. In the simulations, we found that isolated skyrmions can be generated and stabilized in the proposed heterostructures with square-shaped or circle-shaped capping nano-islands. Moreover, because the inhomogeneous PMA distribution is related to the sizes/geometries of the capping nano-islands, it provides the possibility to create the desired skyrmion structures by using different sizes/geometries of capping nano-islands, which offers a guide for the material synthesis in future studies.

### 3.2. Skyrmion Motion in Racetrack Controlled by Inhomogeneous PMA Distribution

**Figure 3:**
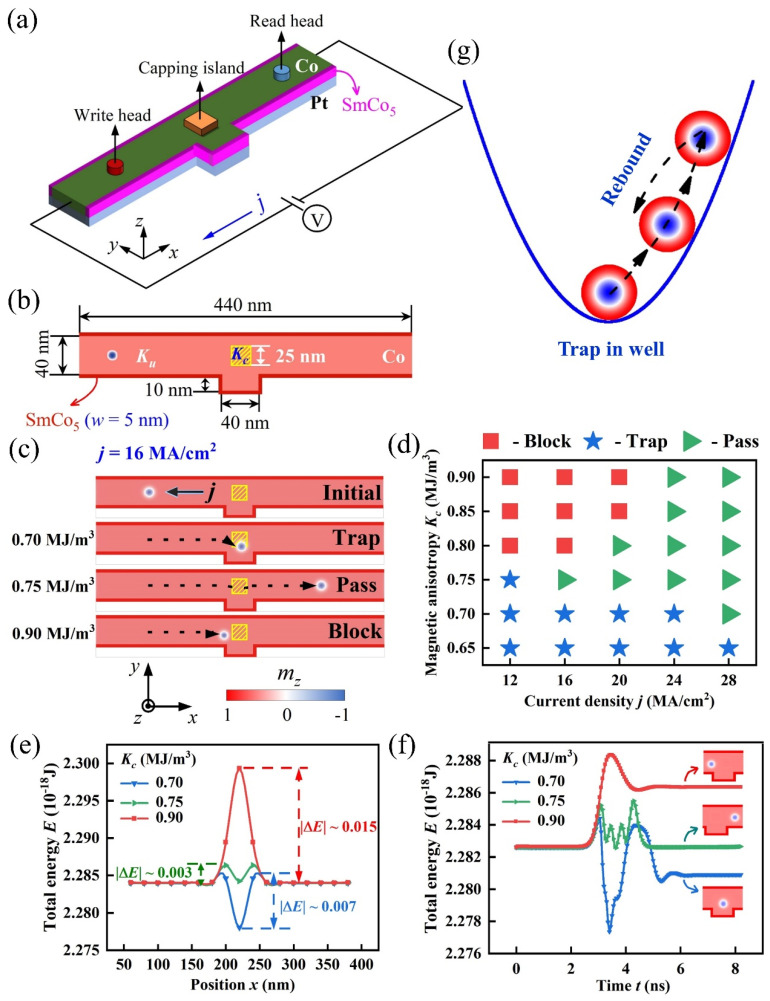
(**a**) Sketch of a racetrack memory with Co/Pt nanotrack. (**b**) Schematic view of nanotrack on the *xy*-plane with inhomogeneous PMA distribution. (**c**) The skyrmion movements in the nanotrack driven by spin current with density *j* = 16 MA/cm^2^. The initial state and the different final states of skyrmion motion (‘block’, ‘trap’ or ‘pass’) at various *K_c_*, with the movement trajectories marked by the dashed lines. (**d**) The state diagram illustrates the condition for the different final states of the skyrmion under various *j* and *K_c_*. (**e**) Plots of the total energy *E* of the whole nanotrack as a function of the position *x* where the skyrmion is located in the nanotrack at various *K_c_*. Here the depth/height of potential well/barrier was denoted as |Δ*E*|. (**f**) Plots of the varying *E* as a function of simulated time *t* for the three typical cases in panel (**c**). Inserts indicate the corresponding final states of skyrmion motion. (**g**) Schematic draw of the skyrmion rebound movement in the potential well for the ‘trap’ state.

In this section, we investigated skyrmion motion in the nanotrack controlled by inhomogeneous PMA distribution, as schematically depicted in Figure 3. The nanotrack in Figure 3a consists of a convex structure, nanotrack edges of high-*K* material SmCo_5_ (colored red, width of *w* = 5 nm), magnetic tunnel junction (MTJ) write head, capping island, MTJ read head and a closed-loop electric circuit. In Figure 3b, the PMA strength (*K_c_*) in nano-island (enclosed by the yellow panel) is variable by the capping materials, and the PMA strength (*K_u_*) for the Co/Pt film (colored pink) is fixed at *K_u_* = 0.8 MJ/m^3^. Here, the high-*K* material SmCo_5_ is rimmed at the upper and lower edges of nanotrack to avoid the undesired annihilation of skyrmions during the motion. This technique had been proposed in previous studies, and it was proven to be effective to shield the skyrmions from annihilation at the nanotrack edge, although the skyrmion drift appears during the motion due to the Magnus force [43,44]. In the simulation, the typical parameters for SmCo_5_ were used as [43,44]: the spontaneous magnetization M_s_ = 840 KA/m, exchange constant *A_ex_* = 12 pJ/m, DMI constant *D* = 0.1mJ/m^2^, PMA strength *K_e_* = 17.1 MJ/m^3^. In addition, a special convex structure with a small size of 40 nm × 10 nm was designed in the nanotrack, because it was essential for the implement of the logic AND operation of ‘1 + 1 = 1’ based on two interacting skyrmions dynamics, as will be elucidated below.

In the simulations, we first created the skyrmion by injecting a local vertical spin-polarized current through the write head (see Figure 3a). After that, the skyrmion moves along the *x*-direction in nanotrack driven by the in-plane spin current. The skyrmion can be detected if it arrives below the MTJ reader head region, by utilizing the tunnel magneto-resistance (TMR) effect [32,33,45]. Figure 3c shows the skyrmion movement driven by in-plane spin current with current density *j* = 16 MA/cm^2^ in the nanotrack. We found that the skyrmion can pass through the capping nano-island at a moderate magnetic anisotropy *K_c_* = 0.75 MJ/m^3^, while it is blocked at the left side of the capping nano-island at relatively large anisotropy *K_c_* = 0.90 MJ/m^3^ and is trapped inside the capping region at relatively small anisotropy *K_c_* = 0.70 MJ/m^3^. These three final skyrmion states are abbreviated to ‘pass’, ‘block’ and ‘trap’. Further simulations generated the phase diagram for the final states of skyrmion after it reaches the capping region under various driving current density *j* and magnetic anisotropy *K_c_*, as summarized in Figure 3d. It was noted that the final skyrmion states are determined by *j* and *K_c_* in the capping island. In the diagram, the ‘trap’ states occupy the region of small *K_c_*, while the ‘block’ states dominate the region of large *K_c_* and small *j*, and the ‘pass’ states distribute in the rest region.

In fact, one may consider the final states of the skyrmion as a result of the competition between *j* and *K_c_*. To further understand these results, we investigated the detailed process of the skyrmion movements in the three typical cases of Figure 3c, from the viewpoint of energy competition. We first generated the isolated skyrmion at different position *x* in the nanotrack by injecting a local vertical spin current, and then calculated the total energy *E* over the whole nanotrack for skyrmion at different position *x*, as the *E*-*x* curves plotted in Figure 3e under various *K_c_*. For this, one may consider the *E*-*x* curves to approximately represent the potential energy of nanotrack along *x* direction at various *K_c_*. Therefore, the capping island with *K_c_* = 0.70 MJ/m^3^ acts as a deep potential well with depth of |Δ*E*| = 0.7 × 10^−20^ J, while the capping island with *K_c_* = 0.75 MJ/m^3^ and *K_c_* = 0.90 MJ/m^3^ generate the potential barrier with small height of |Δ*E*| = 0.3 × 10^−20^ J and large height of |Δ*E*| = 1.5 × 10^−20^ J, respectively.

In Figure 3f, the varying *E*-*t* curves implies that the skyrmion tries to escape from the attraction of the potential well at *K_c_* = 0.70 MJ/m^3^, while it is failed due to the spin current density *j* = 16 MA/cm^2^ here is relatively small and not sufficient to pull the skyrmion out of the potential well. Thus, the skyrmion first climbs to a position with relatively high *E* in the potential well, while rebounds back to a position with relatively low *E* in the potential well, and it is finally trapped inside the potential well, as schematically drawn in Figure 3g. In fact, the skyrmion would like to stay at the lowest *E* in the potential well in the absence of driving current, making the ‘trap’ state of skyrmion quite energetically stable as merit in storing the skyrmionic bits.

On the other hand, the spin current density *j* = 16 MA/cm^2^ can help the skyrmion pass through the low potential barrier at *K_c_* = 0.75 MJ/m^3^, while it fails to drive the skyrmion to pass through the high potential barrier at *K_c_* = 0.90 MJ/m^3^, and the skyrmion finally is blocked at the left side of the capping region. Therefore, these results suggest that the height/depth of potential barrier/well can be mediated by *K_c_* in the capping region, which allows controlling the final ‘pass’, ‘block’ or ‘trap’ state of the skyrmion with suitable values of *j* and *K_c_*.

### 3.3. Skyrmion-based Logic Devices with AND and NOT Operation

Considering the skyrmion motion behaviors in inhomogeneous PMA distribution, we exploited their possible applications in functional spintronic devices. In this section, we first utilized the final ‘trap’ state of skyrmion for devising a skyrmion-based nanotrack device of logic AND operation, taking account of the complicated dynamical behaviors of two interacting skyrmions movements in nanotracks.

**Figure 4:**
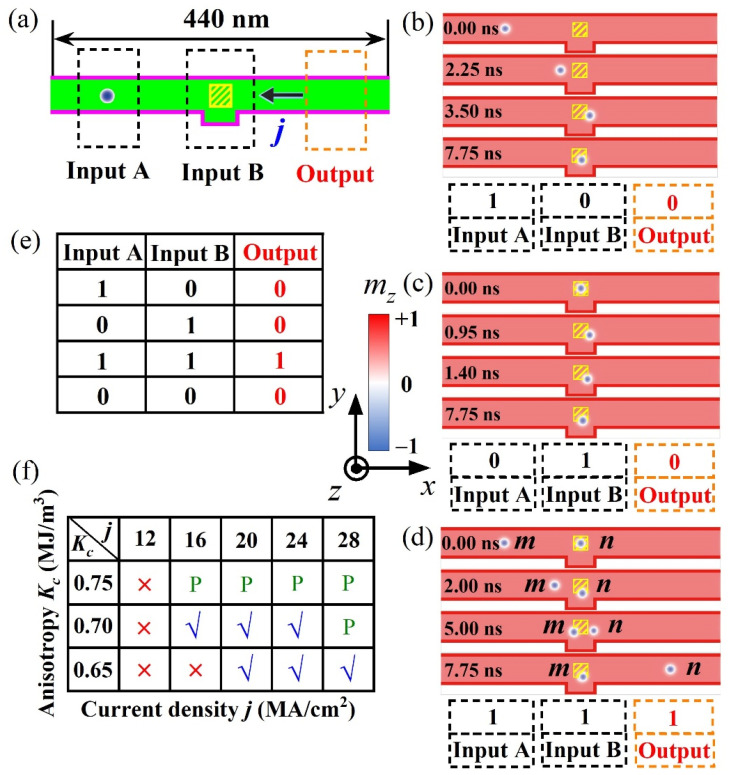
Layout for logic device and logic AND operation. (**a**) Sketch of logic device consisting of three main regions, i.e., ‘Input A’, ‘Input B’ and ‘Output’. (**b**,**c**) The movement of an isolated skyrmion in panel (**b**) ‘Input A’ or (**c**) ‘Input B’ driven by in-plane spin current, achieving the logic AND operation of panel (**b**) ‘1 + 0 = 0’ or (c) ‘0 + 1 = 0’. (**d**) The movements of two skyrmions located in ‘Input A’ and ‘Input B’ driven by in-plane spin current, realizing the logic AND operation of ‘1 + 1 = 1’. All the AND operations in panels (**b**–**d**) are carried out under constant *K_c_* = 0.7 MJ/m^3^ and spin current density *j* = 16 MA/cm^2^. The size of the square-shaped capping island in the ‘Input B’ region is 25 nm × 25 nm. (**e**) The table for the logic AND operations. (**f**) The diagram shows the parameter window for realizing the AND operations. ‘√’ and ‘×’ represent the capability and inability of realizing the AND operations. ‘P’ denotes the ‘pass’ state for the isolated skyrmion when it runs through the potential well, which also fails to meet the requirement of AND operations.

Figure 4 shows the sketch of logic device and operation of logic AND functions. In Figure 4a, the logic device is mainly composed by three regions, that are the ‘Input A’, ‘Input B’ and ‘Output’ regions. Both ‘Input A’ and ‘Input B’ regions were used as the input terminals of skyrmion information carriers, and the ‘Output’ region was used as the output terminal of the skyrmion information carriers. Magnetic anisotropy in the ‘Input A’ region was set as a constant *K_u_* = 0.8 MJ/m^3^, and PMA for the capping island in ‘Input B’ region was fixed as *K_c_* = 0.7 MJ/m^3^ in Figure 4b–d. In the implement of logic operations, the existence or absence of a skyrmion in these regions represents the data bit ‘1’ or ‘0’, respectively.

In Figure 4b, we first created an isolated skyrmion in ‘Input A’ region by injecting vertical spin current. Then the isolated skyrmion was pulled from ‘Input A’ region at *t* = 0 ns to ‘Input B’ region at *t* ~3.5 ns, by an in-plane driving spin current. Finally, the isolated skyrmion stays at ‘Input B’ due to the restraint of potential well in ‘Input B’ region, which attains the logic AND operation of ‘1 + 0 = 0’ in this process. In Figure 4c, an isolated skyrmion was generated in ‘Input B’ region by injecting vertical spin current, while the subsequent in-plane spin current does not provide sufficient energy for helping the isolated skyrmion to escape from the attraction of potential well, and the skyrmion thus becomes stable in the potential well. In this process, the logic AND operation of ‘0 + 1 = 0’ is achieved.

In Figure 4d, two skyrmions were first created in ‘Input A’ and ‘Input B’ regions, denoted as skyrmion *m* (left) and *n* (right), respectively. Then, the skyrmion *m* moves towards the ‘Input B’ region driven by the in-plane spin current and picks up kinetic energy [46,47,48]. In this process, skyrmion *n* keeps nearly stationary in the ‘Input B’ region until the skyrmion *m* is close to the ‘Input B’ region at *t* ~2 ns. After that, as the kinetic energy of skyrmion *m* is larger than that of skyrmion *n*, skyrmion *m* pushes skyrmion *n* away from the ‘Input B’ region via the repulsion between these two skyrmions [49,50]. At last, the skyrmion *n* acquires some kinetic energy which enables it to escape from the energy well and it then runs along the *x*-axis. While skyrmion *m* losses some kinetic energy, it is consequently captured in the potential well and finally ceases movement in ‘Input B’ region. During this process, the logic AND operation of ‘1 + 1 = 1’ is attained, and each skyrmion undergoes complicated dynamics. In the simulations, we found that the convex structure in the racetrack is essential for the implementation of logic operation of ‘1 + 1 = 1’, because the skyrmion *m* is always blocked at the left side of the capping region without the designed convex structure, and in this case it is hard for pushing the skyrmion *n* out of the capping region by the repulsion between two skyrmions.

Based on the simulation results and analyses above, all the AND operations (see Figure 4e) are well implemented in such a logic device, noting that the operation of ‘0 + 0 = 0’ is automatically satisfied. In addition, regarding the repeated use of AND operation in the logic device, one only need to reset the skyrmionic bit in the device by annihilating the skyrmion staying in the ‘Input B’ region after the AND operation. For achieving this operation, we found that the skyrmion can be well annihilated by injecting a vertical spin current with the opposite direction in contrast to that used for the nucleation of skyrmion. Moreover, further simulations generated the diagram for the condition windows of realizing the AND operations in Figure 4f. Comparing the conditions of ‘√’ and ‘×’ (i.e., the capability and inability of realizing AND operations), one may find that the conditions for realizing the AND operations are relative low *K_c_* for creating a potential well and also sufficient large *j* for guaranteeing that the skyrmion *m* will gain enough kinetic energy for pushing the skyrmion *n* out of ‘Input B’ region. In the simulations, it was found that the inability of realizing AND operations (i.e., ‘×’ region) is mainly attributed to the failure in attaining the operation of ‘1 + 1 = 1’, because of the relatively low kinetic energy of skyrmion *m*.

**Figure 5:**
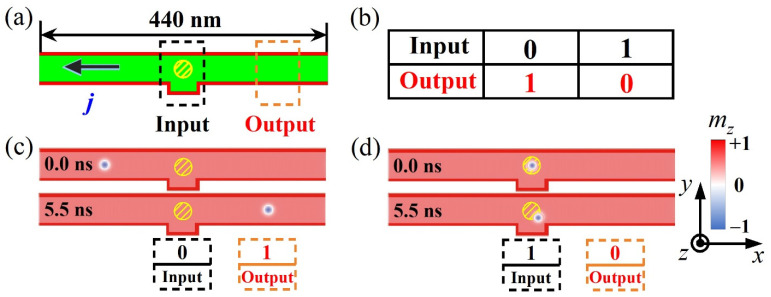
Layout for logic device and logic NOT operation. (**a**) Sketch of logic device consisting of ‘Input’, and ‘Output’ regions. (**b**) The table for the logic NOT operations. (**c**,**d**) The moving isolated skyrmion in the nanotrack achieves the operations of 0 → 1 and 1 → 0. All the operations are carried out under constant *K_c_* = 0.7 MJ/m^3^ and spin current density *j* = 20 MA/cm^2^. Here the diameter of the circle-shaped capping nano-island in the ‘Input’ region is 25 nm.

Besides working as AND logic operations, nanotracks with various geometries and sizes of capping nano-islands can be extended for exploiting other possible logical functions. We proposed the nanotrack with circle-shaped capping nano-island in Figure 5a, and employed the similar scheme that used in the AND logic operations to nucleate the skyrmion and manipulate its motion in the nanotrack. It can be seen in Figure 5c,d that, the operations of 0 → 1 and 1 → 0 are attained in the nanotrack, which achieves the logic NOT operation (see Figure 5b). Therefore, the logic AND and NOT operations were successfully realized in the proposed racetrack logical devices.

## 4. Conclusions

Before concluding this work, we briefly discuss the multifunctional logic devices and thermal stability of the magnetic skyrmions. It is noted that the multiple logic operations including AND, OR, NAND, NOR, XOR and XNOR had be implemented in the VCMA controlled logical devices, which usually demands the transport of skyrmions in the multi-channel and multi-network circuit [34,35,36,37]. However, the nanotrack studied here is only a simple single-channel circuit, which can achieve a few special logic functions. Therefore, this suggests the opportunity to explore the other logical devices in the multi-channel and multi-network circuits, in which the nanotracks with AND and NOT logic functions may be employed as basic circuit elements. This would be an interesting challenge for our further studies.

On the other hand, all simulations in present study were carried out under zero temperature condition based on OOMMF calculation, in which the thermal effect is neglected. However, the thermal stability of magnetic skyrmions is also a crucial issue for a detailed understanding of their underlying physical properties. Besides, the practical applications of the spintronic devices demand operation at room temperature or above. In this regard, previous studies [13,51,52,53,54] demonstrated that skyrmions in a variety of Co/Pt-based multilayers (e.g., Pt/Co/Ta and Pt/Co/MgO) can be stable at room temperature. These provide us an enlightened approach to enhance the thermal stability of skyrmions in the Co/Pt systems in the further studies.

In summary, we have investigated the modulation of skyrmion structures and their motion behaviors in ultrathin Co/Pt heterostructures with inhomogeneous PMA from the capping nano-island, by means of micromagnetic simulation. The inhomogeneous PMA distribution can behave as tunable nanoscale potential barriers/wells, which are able to effective modulate the size and shape of isolated skyrmion in a square-shaped thin-film heterostructure. In addition, the inhomogeneous PMA can manipulate the skyrmion motion behaviors in a specifically designed racetrack structure, and enables different motion modes, i.e., blocking, passing, and trapping. Based on these observations, a set of skyrmion racetrack logical devices were proposed, which are able to successfully achieve the logic AND and NOT operations, by taking into account of the complicated dynamical behaviors of two interacting skyrmion movements in nanotracks. The present study demonstrates a new pathway for developing new spintronic devices based on manipulation of skyrmion motion via inhomogeneous PMA. Similar ideas may be extended to explore other functional devices using complex geometries of nano-island or tunable VCMA gates in the multi-channel and multi-network circuits.

## Data Availability

All the data present in this paper will be made available upon reasonable request. Please contact the corresponding author for further information.
